# Diagnostic biomarker for type 2 diabetic peripheral neuropathy via comprehensive bioinformatics analysis

**DOI:** 10.1111/1753-0407.13506

**Published:** 2023-11-29

**Authors:** Xiaoyu Chen, Qingquan Liu, Niyao Chen, Jiangxin Ma, Xiaohong Wu, Haibin Zhang, Liying Yu, Huibin Huang

**Affiliations:** ^1^ Department of Endocrinology The Second Affiliated Hospital of Fujian Medical University Quanzhou China; ^2^ Department of Cardiology The Second Affiliated Hospital of Fujian Medical University Quanzhou China; ^3^ Central Laboratory The Second Affiliated Hospital of Fujian Medical University Quanzhou China

**Keywords:** bioinformatic analysis, immune gene, immune infiltration, peripheral neuropathy, Type 2 diabetes

## Abstract

**Background:**

Diabetic peripheral neuropathy (DPN) is a common complication of Type 2 diabetes mellitus (T2DM), which frequently results in disabling neuropathic pain and lower‐limb amputation. The identification of noninvasive biomarkers for DPN may help early detection and individualized treatment of DPN.

**Methods:**

In this study, we identified differentially expressed genes (DEGs) between DPN and the control based on blood‐source (GSE95849) and tissue‐source gene expression profiles (GSE143979) from the Gene Expression Omnibus (GEO) database using limma, edgeR, and DESeq2 approaches. KEGGG and GO functional enrichments were performed. Hub genes and their correlation with infiltrating immune cells were analyzed. Real‐time quantitative polymerase chain reaction (RT‐qPCR) was used to quantify hub gene expression.

**Results:**

In total, 144 DEGs between DPN and the control were identified. Functional enrichment revealed that the DEGs were mainly enriched in immune‐related pathways like the Fc epsilon receptor Ig signaling pathway. By protein–protein interaction (PPI) network analysis, *FCER1G*, *SYK*, *ITGA4*, *F13A1*, *MS4A2*, and *PTK2B* were screened as hub genes with higher expression in DPN patients, among which half were immune genes (*FCER1G*, *PTK2B*, and *SYK*). RT‐qPCR demonstrated that mRNA expression of *FCER1G*, *PTK2B*, and *SYK* was significantly increased in patients with DPN compared with both diabetic nonperipheral neuropathy (DNN) and normal subjects. The area under the receiver operating characteristic (ROC) curve of *FCER1G*, *PTK2B*, and *SYK* was 0.84, 0.81, and 0.73, respectively, suggesting their great advantages as diagnostic biomarkers to predict the progression of neuropathy in T2DM. Further analysis indicated that the expression of *FCER1G*, *PTK2B*, and *SYK* was negatively correlated with the cell proportion of significantly altered resting natural killer cells, T follicular helper cells, and activated mast cells, but positively correlated with monocytes.

**Conclusions:**

Our findings demonstrated *FCER1G*, *PTK2B*, and *SYK* are potential diagnostic biomarkers and therapeutic targets for DPN, which provides new insight into DPN pathogenesis and therapies.

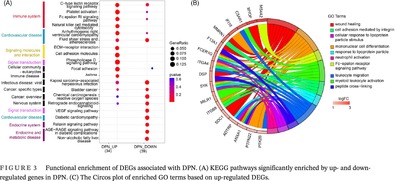

## Graphical abstract

**Highlights**

- Differentially expressed genes between diabetic peripheral neuropathy (DPN) and normal controls are mainly enriched in immune‐related pathways.
- Three immune genes—*FCER1G*, *PTK2B*, and *SYK*—were identified as diagnostic and prognostic biomarkers for DPN.
- Gene expression of *FCER1G*, *PTK2B*, and *SYK* was negatively correlated with the cell proportion of resting natural killer cells, T follicular helper cells, and activated mast cells, but positively correlated with monocytes.

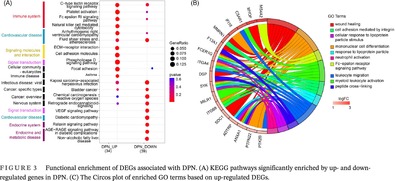

## 1 INTRODUCTION

Diabetes mellitus (DM) is a chronic metabolic disorder characterized by persistent hyperglycemia that is hugely escalating worldwide.^1^ Type 2 diabetes mellitus (T2DM) accounts for approximately 90% of all cases of diabetes. From 1980 to 2014, the prevalence of all types of DM nearly doubled from 4.7% to 8.5%.^2^ The global prevalence of diabetes was roughly 463 million people in the year 2019, and it is estimated to rise by 25% in 2030 and 51% in 2045.^3^ DM is a worldwide health burden since the annual global health cost for DM is currently about US$760 billion and is expected to rise to US$825 billion by 2030.^3^

Diabetic peripheral neuropathy (DPN) is one of the most common complications of DM, affecting up to 50% of patients with diabetes.^4^ It is characterized by peripheral nerve damage caused by persistently high blood glucose levels. DPN is the main precipitating factor for diabetic foot complications, such as ulceration and Charcot neuroarthropathy, which usually result in disabling neuropathic pain and lower‐limb amputation. Amputations in DM patients impose a great economic burden, a devastating impact on quality of life, and a low life expectancy of an average of 2 years.^5^

The progression of DPN is difficult to diagnose and quantify at the asymptomatic stage. Identification of genes related to DPN and its mechanisms will aid in the early detection and personalized treatment of the complication. Despite the clear association between T2DM and DPN, the underlying molecular mechanisms of neurodegeneration are still poorly understood, although some genes and their variants have been reported and attributed to DPN,^6^, ^7^ such as the *CNDP1*, *ELMO1*, and *NOS3* genes.^6^ Vascular endothelial growth factor (VEGF) gene variations were strongly associated with DPN.^8^ The single nucleotide polymorphism (rs4496877) of the *NOS3* gene was observed in patients with DPN.^9^ Interestingly, research revealed that genes associated with DPN varied among populations.^7^ The molecular pathways, including endoplasmic reticulum stress,^10^ increased oxidative stress,^11^, ^12^ mitochondrial dysfunction,^11^ and inflammation,^13^ are linked to DPN progression. However, these studies primarily focused on gene changes in animals, whereas humans may exhibit different expression patterns or responses.

In this study, we investigated marker genes associated with DPN. Based on two Gene Expression Omnibus (GEO) datasets, we uncovered substantially regulated genes in DPN patients. The biological functions of the differentially expressed genes (DEGs) were confirmed using the Kyoto Encyclopedia of Genes and Genomes (KEGG) and Gene Ontology (GO) enrichment. A protein–protein interaction (PPI) network was constructed to screen out six hub genes including Fc Epsilon Receptor Ig (*FCER1G*), Spleen associated Tyrosine Kinase (*SYK*), Integrin Subunit Alpha 4 (*ITGA4*), Coagulation Factor XIII A Chain (*F13A1*), Membrane‐Spanning 4‐Domains A2 (*MS4A2*), and Protein Tyrosine Kinase 2 Beta (*PTK2B*). Additionally, immune infiltrating cells were evaluated and correlated with hub genes. To improve our understanding of the clinical values of the hub genes, we experimentally validated hub genes in clinical specimens and examined their clinical features. Our findings demonstrated that three immune‐related genes (*FCER1G*, *PTK2B*, and *SYK*) were associated with DPN and may serve as potential diagnostic and prognostic biomarkers, which provides new insights into DPN and lays a foundation for molecular mechanism investigation of DPN progression.

## 2 MATERIALS AND METHODS

### 2.1 Data sources

Two independent genome‐wide gene expression datasets of DPN were downloaded from the GEO database: microarray‐based gene expression of blood samples from GSE95849 (GPL22448 platform), along with six healthy individuals (CN1–CN6), six T2DM patients (DM1–DM6), and six DPN patients (DPN1–DPN6)^14^; and RNA sequencing (RNA‐seq)‐based gene expression of tissue samples from GSE143979 (Illumina HiSeq 3000), including eight medial gastrocnemius (MG) muscle samples of the calf as control (MG1–MG8) and seven abductor hallucis (AH) muscle samples of the foot as the case group (AH1–AH7). The probes in the microarray data were converted to the corresponding gene symbols. Expression of the gene with multiple probes was averaged. A list of 2484 immune‐relevant genes was retrieved from the Immunology Database and Analysis Portal (ImmPort, https://immport.niaid.nih.gov).

### 2.2 Identification of DEGs

**FIGURE 1:**
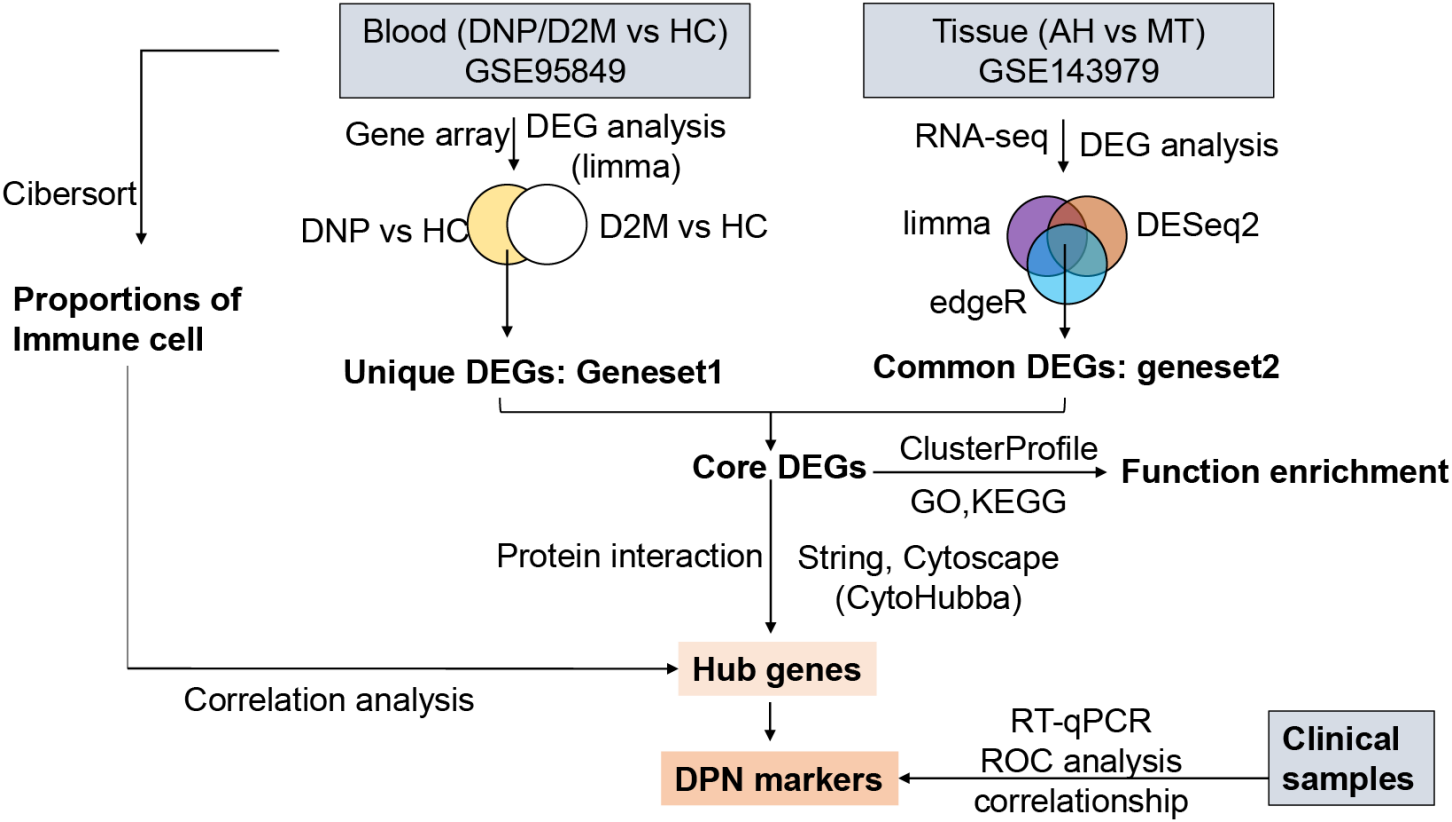
Flow chart of this study.

Multiple methods were employed for identifying DEGs to accurately determine dysregulated genes associated with DPN (Figure 1). Genes that were not detected in more than 75% of samples were filtered out for further analysis. The “limma” R package was used to compare the microarray‐based gene expression between the DPN/T2DM and healthy control (HC) groups of the GSE95849 cohort. DEGs were defined as genes with a *p* value <0.05 and |FoldChange| > 2. DEGs from the DPN‐versus‐HC comparison that eliminated those from the T2DM‐versus‐HC comparison were assumed to be dysregulated genes in the blood of DPN (here referred to as geneset1). Differential expression of RNA‐seq data between the AH and MG sample groups was determined with limma, edgeR, and DESeq2 software. The common DEGs derived by the three methods were defined as the tissue DEGs of DPN (referred to as geneset2). The intersection of geneset1 and geneset2 was regarded as the final DEGs associated with DPN.

### 2.3 Functional enrichment

The KEGG and GO functional enrichment of DEGs were performed by the “ClusterProfiler” R package. The KEGG pathways or GO terms with a *p* value <0.05 were considered significantly enriched. For upregulated or downregulated DEGs, the top 10 enriched pathways or GO terms were displayed.

### 2.4 Diagnostical biomarker screening

To uncover potential diagnostic gene markers for DPN, a PPI network was first constructed using the STRING database (https://www.string-db.org/). Based on the PPI genes, the hub genes were identified using the intersection of the top 10 genes resulting from five topological analysis approaches (Maximal Clique Centrality [MCC], Edge Percolated Component [EPC], Closeness, Degree, and Betweenness) of CytoHubba in the Cytoscape plug‐in. Biomarkers for diagnosis and experimental verification were determined as genes shared between hub genes and the list of immune‐relevant genes.

### 2.5 Profiling of infiltrating immune cells and their correlation with diagnostical biomarkers

The abundance of 22 types of immune cells in each blood sample was estimated using the CIBERSORT algorithm. The differences in immune cell proportion were compared between the control group and the case groups using the Wilcoxon signed rank sum test. The Spearman correlations between immune cell proportion and the expression of diagnostical biomarkers were calculated using the R “ggpubr” package and visualized using the “ggplot2” package.

### 2.6 Recruitment of participants

A cohort of patients who were diagnosed with DPN was used as case group (DPN, *n* = 41). A healthy donor cohort (normal, *n* = 52) and a diabetic non‐neuropathy cohort (DNN, *n* = 41) were used as control groups. The blood samples and clinical factors of the participants were collected. The blood samples were provided by the Second Affiliated Hospital of Fujian Medical University (Quanzhou, Fujian, China) with the approval of the institutional research board and the donors' consent (Date 30‐7‐2020 / No.79). The procedures followed in this study were in accordance with the ethical standards of the relevant institutional policies.

### 2.7 RNA extraction and real‐time quantitative polymerase chain reaction

Human peripheral blood mononuclear cells (PBMCs) were isolated for real‐time quantitative polymerase chain reaction (RT‐qPCR). Total RNA was isolated utilizing TRIzol (Invitrogen, California, USA) and reverse‐transcribed with an mRNA reverse transcription kit (Takara, Japan). Specific RT‐qPCR primers were designed to quantify the mRNA expression of biomarker genes. All primers were synthesized by Sangon Biotech (Shanghai, China), and their sequences are listed in Table S1. Relative quantification was calculated using the 2^−ΔΔCt^ method.

### 2.8 Validation of prognostic values of gene markers

The overall prognostic evaluation of the diagnostic markers was accomplished via receiver operating characteristic (ROC) curve analysis with the “survivalROC” R package. The area under the curve (AUC) was calculated to assess the diagnostic value of the marker.

### 2.9 Statistical analysis and graphing

For pairwise comparisons, the Wilcoxon signed rank sum test was used. Statistical analysis and plotting were performed using R software. The heatmap of gene expression was visualized using the R package “pheatmap.” Principal component analysis (PCA) was performed using the “prcomp” R package.

## 3 RESULTS

### 3.1 Characterization of genes associated with DPN

**FIGURE 2:**
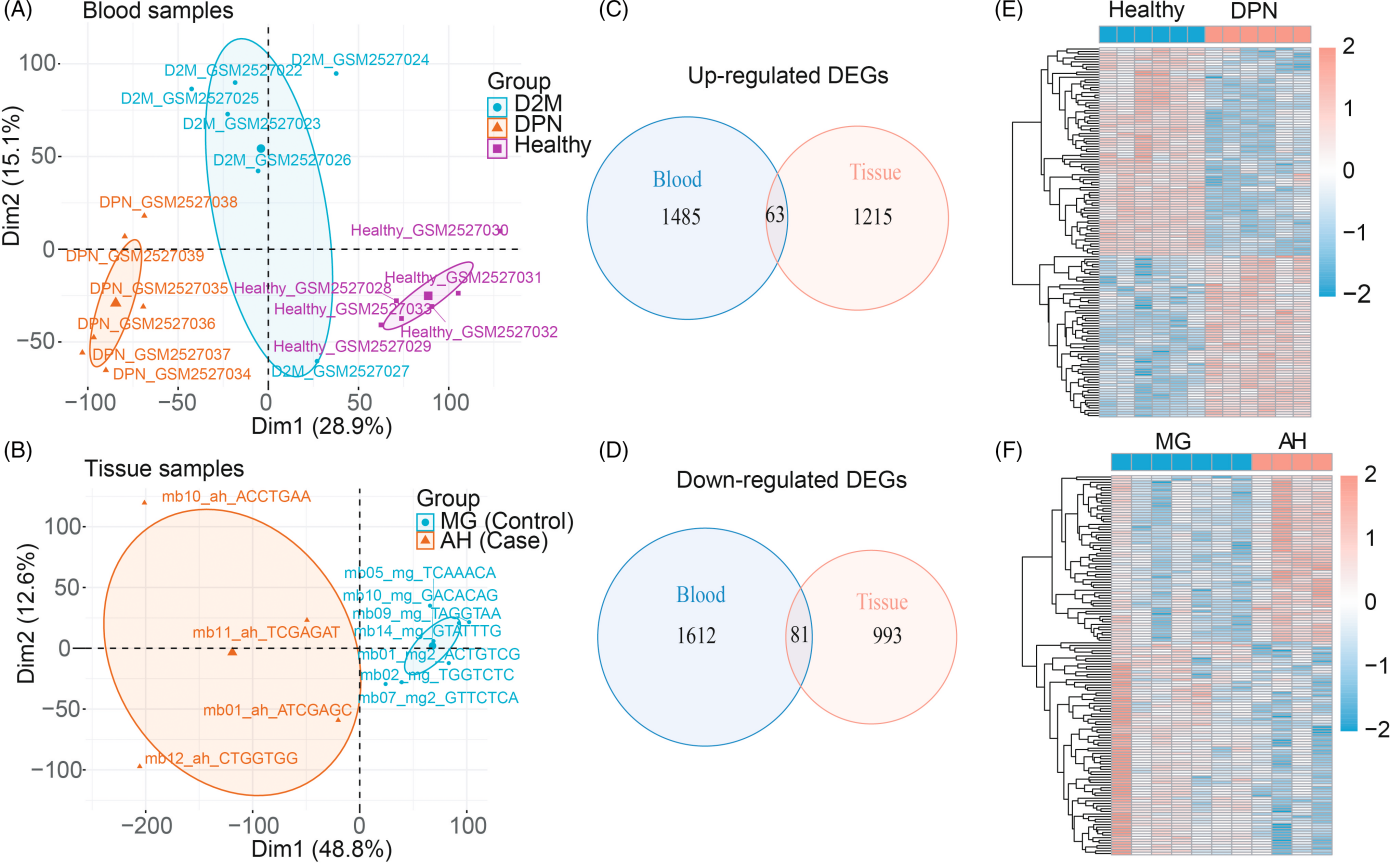
Identification of DEGs associated with DPN. Principal component analysis (PCA) of blood samples from the GSE95849 dataset (A) and tissue samples from the GSE143979 dataset (B). The Venn diagram of genes that were up‐regulated (C) and down‐regulated genes (D) in both blood and tissue samples. (D) Heatmap of the expression of DEGs commonly intersected in blood (E) and tissue samples (F).

Figure 1 displays the analysis flowchart that we used for this research. PCA revealed that samples differed considerably among the three groups (T2DM, DPN, and HC) from the GSE95849 blood dataset and were suitable for gene expression comparisons within groups (Figure 2A). As a result, 4508 and 1772 DEGs were screened from DPN‐versus‐HC and T2DM‐versus‐HC comparisons in the blood samples, respectively. Except for the DEGs from T2DM‐versus‐HC, 3241 (geneset1) out of 4508 DEGs, including 1693 upregulated and 1548 downregulated DEGs, were exclusively found in DPN patients compared to healthy individuals. In the GSE143979 tissue dataset, the PCA results showed that four AH samples (three outliers were excluded) and seven MG samples were grouped apart from one another. These samples had a high percentage of PC1, accounting for 48.8% (Figure 2B). The edgeR, DESeq2, and limma algorithms generated 3923, 3459, and 3201 DEGs between the AH and MG samples, respectively, contributing to a total of 2352 common DEGs (geneset2) with 1278 upregulated and 1074 downregulated genes. The intersection of upregulated or downregulated DEGs from the blood (geneset1) and tissue samples (geneset2) yielded 144 DEGs, with 63 upregulated genes and 81 downregulated genes (Figure 2C–F).

### 3.2 Functional pathway of DEGs involved in DPN regulation

**FIGURE 3:**
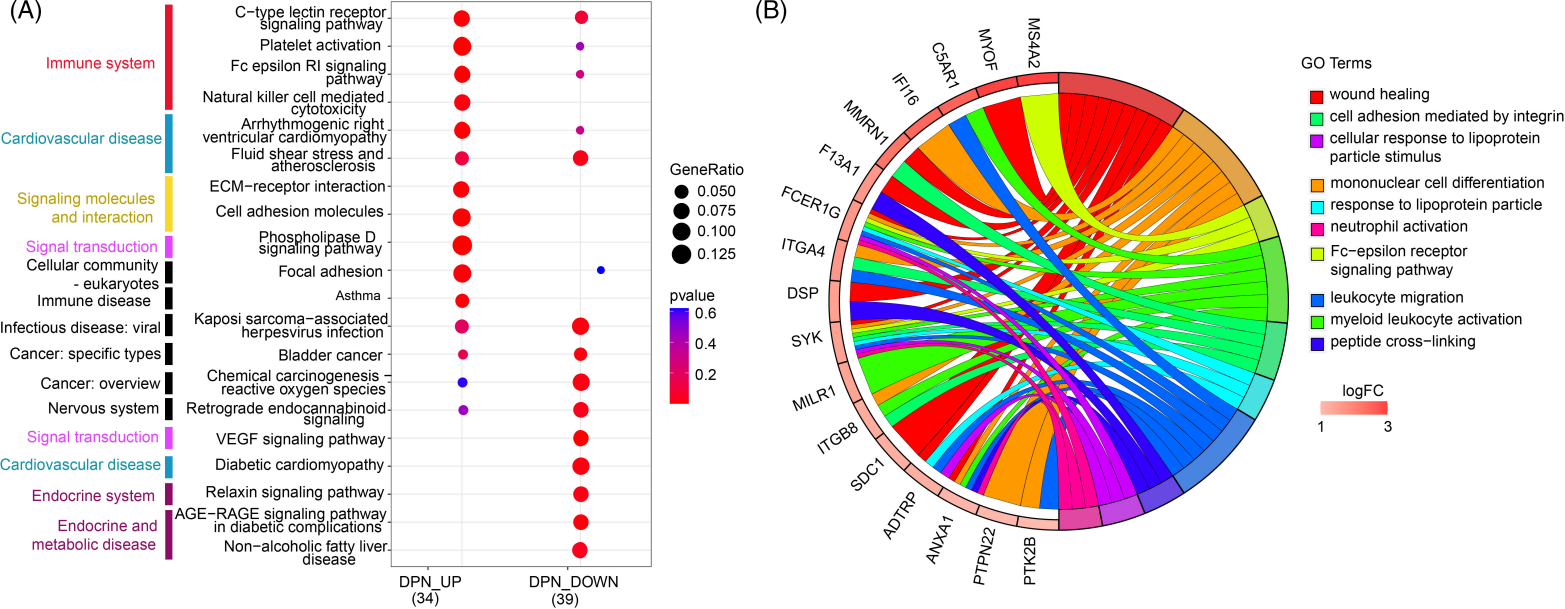
Functional enrichment of DEGs associated with DPN. (A) KEGG pathways significantly enriched by up‐ and down‐ regulated genes in DPN. (B) The Circos plot of enriched GO terms based on up‐regulated DEGs.

To determine the biological function of DEGs in DPN, we carried out KEGG enrichment on the upregulated and downregulated DEGs separately. We discovered that the significantly upregulated and downregulated genes commonly participate in immune system (Level 2 category) pathways, such as the C‐type lectin receptor signaling pathway, platelet activation, and Fc epsilon receptor Ig (RI) signaling pathway, and cardiovascular disease (Level 2 category), including arrhythmogenic right ventricular cardiomyopathy, fluid shear stress, and atherosclerosis (Figure 3A). In addition, the enhanced genes in DPN are enriched in natural killer (NK) cell‐mediated cytotoxicity of the immune system (Level 2 category); extracellular matrix (ECM)‐receptor interaction, and cell adhesion molecules of signaling molecules and interaction (Level 2); and phospholipase D signaling pathways of signal transduction (Level 2 category). The significantly decreased genes in DPN were also uniquely enriched in endocrine and metabolic disease (Level 2 category), which included the AGE–RAGE (advanced glycation end product–advanced glycation end product receptor) signaling pathway in diabetic complications, and nonalcoholic fatty liver disease; the relaxin signaling pathway in the endocrine system (Level 2 category); diabetic cardiomyopathy of cardiovascular disease (Level 2 category); and the VEGF signaling pathway in signal transduction (Level 2 category) (Figure 3A).

To identify markers for DPN, we focused on the 63 upregulated DEGs and carried out GO enrichment on the increased genes in DPN. The top 10 enriched pathways were found to be 50% immune related, including mononuclear cell differentiation, neutrophil activation, Fc epsilon RI signaling pathway, leukocyte migration, and myeloid leukocyte activation (Figure 3B). Additionally, a total of 17 enhanced DGEs take part in the top 10 enriched GO categories. Among them, *FCER1G* and *SYK* play roles in more than 80% of the top 10 pathways.

### 3.3 Identification of hub genes

**FIGURE 4:**
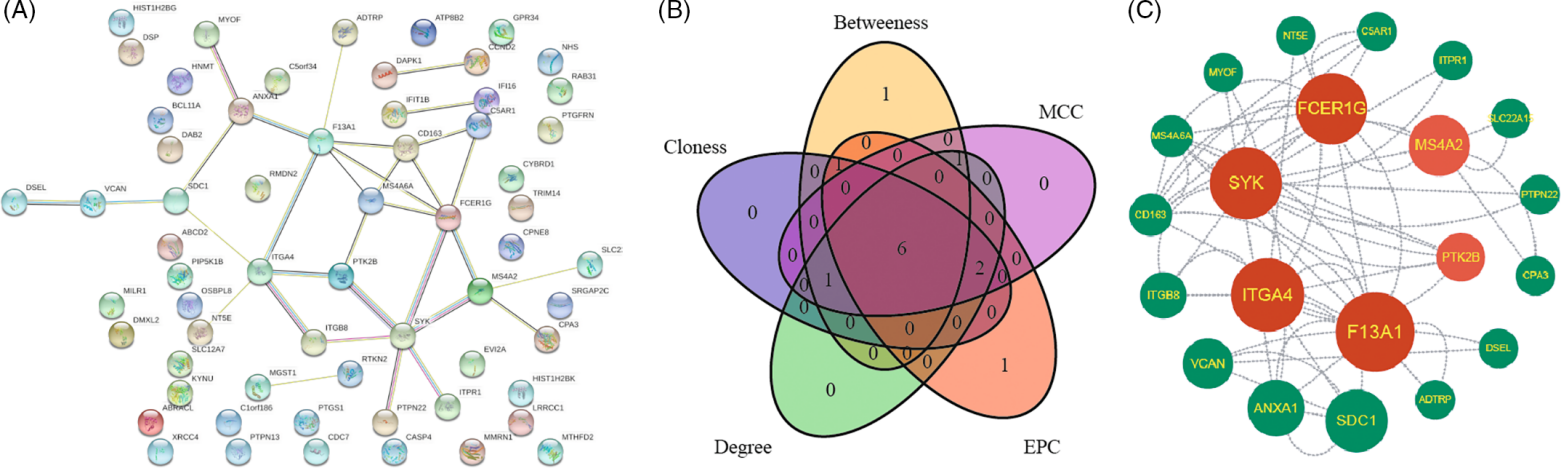
Identification of hub genes based on the protein‐protein interactions (PPI) network of differentially expressed genes (DEGs). (A) The PPI network of up‐regulated DEGs constructed by String. (B) Six hub gene revealed by Venn diagram of the top 10 hub genes screened by MCC, EPC, Closeness, and Betweenness approaches with CytoHubba plug‐in of Cytoscape. (C) The network formed by the 21 increased DEGs and interactions between the six hub genes and other genes.

Protein interaction was estimated using the PPI network constructed by String. The PPI network of the 63 upregulated genes revealed a network of 21 gene interactions (Figure 4A). The hub genes were then selected using five algorithms of Cytohubba plug‐ins in Cytoscape. Six genes were thereby commonly targeted by the Betweenness, MCC, Closeness, Degree, and EPC methods (Figure 4B). The six genes, which played the most important roles in the network of the 21 upregulated DEGs, were *FCER1G*, *SYK*, *ITGA4*, *F13A1*, *MS4A2*, and *PTK2B* (Figure 4C). Among the six hub genes, *FCER1G*, *SYK*, and *PTK2B* were immune genes.

### 3.4 Immune cell composition in DPN

**FIGURE 5:**
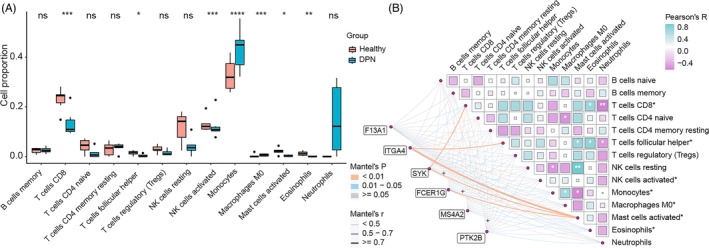
Immune infiltration and their relationship with the hub gene expression in the blood samples. (A) Differences in the proportion of 13 immune cells detected by CIBERSORT. (B) Relationship between the proportion of immune cells and the expression of the hub genes.

It was implied by the GO enrichment of upregulated genes that immune cells or pathways were associated with DPN. We examined immune infiltration in DPN and their relationship with the hub genes to figure out more about immune function in DPN. The results indicated that 13 types of 22 immune cells were detected in the blood samples (Figure 5A). The proportion of CD8 T cells, T follicular helper cells, activated NK cells, activated mast cells, and eosinophils were considerably lower in DPN patients compared to healthy individuals, while monocytes and M0 macrophages were more abundant in patients with DPN than the healthy. Besides, the Pearson test showed that the cell proportion of CD8 T cells was negatively correlated with the cell proportion of neutrophils, but positively correlated with eosinophils. T follicular helper cells were negatively correlated to neutrophils, but positively correlated to eosinophils and activated mast cells. Additionally, we assessed the relationship between the gene expression of the six hub genes and the proportion of immune cells (Figure 5B). Six hub genes exhibited a negative correlation with the dramatically altered cells of activated mast cells. Gene expression of *FCER1G*, *SYK*, and *PTK2B* was favorably correlated with monocytes, but negatively correlated with resting NK cells and T follicular helper cells.

### 3.5 Characterization of clinical value of hub genes

**FIGURE 6:**
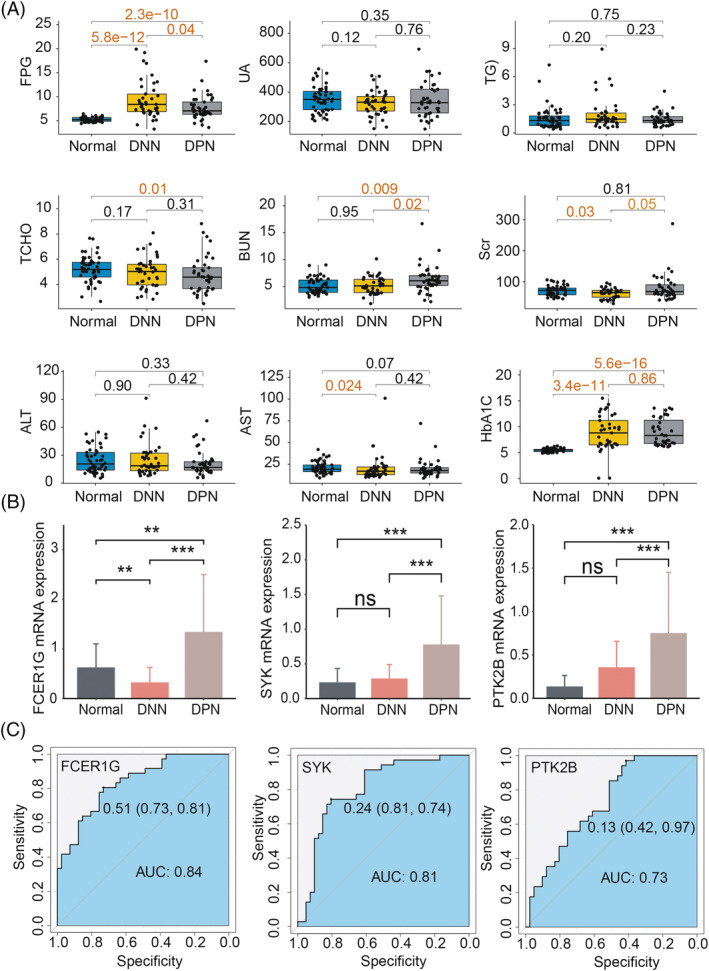
Characteristics of clinical factors and features of hub genes in DPN. (A) Differences of the main clinical factors between normal (*n* = 52), DNN (*n* = 41) and DPN (*n* = 41) groups. The orange digital marks *p*‐value <0.05. (B) The mRNA expression of FCER1G, SYK, and PTK2Bin the normal, DNN and DPN groups quantified by RT‐qPCR. (C) Receiver operating characteristic (ROC) analysis of FCER1G, SYK, and PTK2B for DPN.

To confirm the clinical relevance of the immune‐related hub genes (*FCER1G*, *SYK*, and *PTK2B*) in DPN, 52 healthy individuals, 41 patients with DNN, and 41 patients with DPN were recruited for clinical sample analysis. We found that the clinical factors fasting plasma glucose (FPG), blood urea nitrogen (BUN), and glycosylated hemoglobin (HbA1c) levels were markedly higher in DPN patients (*p* < 0.05) compared with both normal individuals and DNN patients, while total choline (TCHO) was significantly lower (*p* < 0.05) in the DPN group than the normal controls (Figure 6A). Gene expression of *FCER1G*, *SYK*, and *PTK2B* was significantly enhanced in patients with DPN compared with both the HC and DNN patients (*p* < 0.05) (Figure 6B), which is consistent with transcriptomic data. *FCER1G* exhibited a markedly decreased expression in patients with DNN compared with the normal control, while *SYK* and *PTK2B* had no significant differences between DNN patients and the healthy individuals. ROC analysis showed that the AUC of *FCER1G*, *PTK2B*, and *SYK* was 0.84, 0.81, and 0.73, respectively, suggesting good performance of the three immune genes in evaluating DPN (Figure 6C).

## 4 DISCUSSION

DPN frequently leads to neuropathic pain, foot ulceration, and ultimately amputation,^15^ affecting up to 50% of people with diabetes.^16^ Uncovering molecular mechanisms underlying DPN pathogenesis and identification of DPN‐specific diagnostic and prognostic markers has great significance for improving the prognosis of patients with DPN. Despite studies implicating that DNA methylation,^17^ mitochondrial dysfunction,^18^ endogenous RNA regulation,^19^ lncRNA–mRNA interaction,^20^, ^21^ and gene regulation in inflammation and immunity‐related pathways^22^ drive DPN onset, the molecular mechanisms underlying DPN progression are still largely unknown, particularly gene regulation in human subjects. In this research, we combined blood‐sourced and tissue‐sourced transcriptomes from GEO datasets of human DPN to profile mRNA‐level changes in molecular mechanisms underlying DPN pathogenesis. The hub genes associated with DPN and their clinical functions in DPN were screened.

We first identified blood‐source DEGs between DPN and healthy individuals (DPN‐blood DEGs) and then excluded those intersecting with DEGs between T2DM patients and the healthy (T2DM DEGs) to obtain blood‐special DPN‐associated DEGs. Subsequently, we used three approaches (limma, edgeR, and DEseq2) to identify tissue‐source DPN DEGs (DPN‐tissue DEGs) by comparing AH samples with more advanced peripheral nerve degeneration and MG samples with the same systemic features of diabetes. Finally, we selected the targeted DEGs by taking the intersection of blood DPN‐special DEGs and DPN‐tissue DEGs. The application of a combination of various source datasets with three DGE identification methods generated a robust set of DEGs associated with DPN.

Previous studies have suggested that immune response plays a crucial role in DPN development.^17^, ^22^ Our findings also demonstrated that the majority of DEGs were enriched in pathways that are immune associated or highly correlated with the pathogenesis of DPN (such as endocrine and metabolic disease and diabetic complications). Among the immune response pathways, Fc epsilon RI signaling was commonly targeted by GO and KEGG enrichment. In the DPN treatment of Tang‐luo‐ning (TLN), a traditional Chinese herbal recipe, Fc epsilon RI signaling was proposed as a targeted pathway of microRNA regulation.^23^ This pathway also consisted of targeted genes regulated by differentially expressed lncRNAs in DPN patients.^24^ The immune‐related ECM‐receptor interaction was reported to be linked to DPN.^17^, ^19^, ^22^

We observed that half (*FCER1G*, *PTK2B*, and *SYK*) of the hub genes were immune genes, and RT‐qPCR confirmed that their expression was significantly enhanced in DPN. Studies have demonstrated that *FCER1G* was upregulated in different stages of the sciatic nerve and dorsal root ganglia (DRG) of diabetic mice^25^ and contributed to the generation of neuropathic pain in rats.^26^ The elimination of activating *FCER1G*‐dependent Fc gamma receptors (FcγRs) reduced nerve injury by altering endoneurial and systemic inflammation.^27^ *SYK* is widely expressed in hematopoietic cells and is involved in a variety of cellular processes by coupling activated immunoreceptors to downstream signaling events. Numerous studies have shown that *SYK* is closely related to diabetes development^28^, ^29^, ^30^ and peripheral nerve injury.^31^, ^32^ Increased *PTK2B* contributed to peripheral nerve regeneration in rats.^33^ It is also a target of an enhancer element—the CLU risk variant—that is associated with pseudoexfoliation and Alzheimer disease.^34^ Together, the regulation of *FCER1G*, *SYK*, and *PTK2B* on the development of nerve system disease suggests that they most likely contribute to T2DM‐induced peripheral neuropathy.

Immunity‐related pathways were associated with DPN. We further quantitatively characterized immune cell composition using CIBERSORTx and analyzed their relationship with hub genes. Seven of the 13 detected immune cells (53.85%) were significantly changed in DPN. Among them, 71.43% (5/7) were decreased in DPN, indicating an overall decreased immune ability in DPN patients. In addition to mediating immunity, NK cells also express nerve growth factor (NGF),^35^ a neurotrophy crucial for the development and survival of neurons.^36^ T follicular helper cells are essential for long‐live plasma cell differentiation, antibody production, and the pathogenesis of diabetic retinopathy.^37^ As reported in DPN patients, the increased proportion of monocytes in DPN probably caused tissue damage via a variety of pathways.^38^ The positive relationship between monocytes suggests that these pathways are most likely regulated by *FCER1G*, *SYK*, and *PTK2B*. Overall, we speculate that *FCER1G*, *SYK*, and *PTK2B* aggravate DPN by promoting the activation of monocytes and inhibiting resting NK cells and T follicular helper cells.

Our analyses revealed that immune pathways, immune cells, and immune genes play vital roles in the pathological process of DPN. Significantly increased FPG, BUN, and HbA1c levels in DPN suggest their important roles in DPN progression. Studies have showed that HbA1c is a diagnostic and prognostic indicator of T2DM^39^, ^40^ and a biomarker for diabetic foot peripheral neuropathy,^41^ and BUN is noticeably elevated in T2DM rats.^42^ Recently, several source‐based types of biomarkers for DPN diagnosis and prevention were identified, including diagnostic factors from skin punch biopsies,^43^ serum,^44^ and plasma,^45^ and gene biomarkers such as toll‐like receptor 4 (TLR4) and tumor necrosis factor alpha (TNF‐α).^46^ Compared to skin punch biopsies with invasive injury, gene biomarkers and serum and plasma factors are almost harmless. Among them, gene biomarkers displayed the highest AUC (avg. AUC = 0.82) compared with serum (avg. AUC = 0.73) or plasma factors (avg. AUC = 0.77) (Table S2), indicating a higher reliability in predicting disease. The good performance of immune genes *FCER1G*, *SYK*, and *PTK2B* in distinguishing DNN and DPN samples suggests that they are reliable diagnostic and prognostic markers and potential therapeutic targets of DPN.

In this study, we applied bioinformatics analyses and clinical sample verification to characterize potential molecular markers of DPN. The identified markers may provide a new noninvasive, easy‐to‐detect and highly sensitive diagnostic approach for DPN. Additionally, they represent promising therapeutic targets warranting further investigation. This work also establishes a valuable foundation for understanding the pathogenesis of DPN. However, there are several limitations. Due to the small sample size, the transcriptional regulation associated with DPN requires validation and further exploration in larger cohorts, despite the fact that we utilized blood‐sourced and tissue‐sourced transcriptomes and various bioinformatic approaches to uncover DPN‐associated genes and pathways. Furthermore, elucidating the molecular mechanisms of immune genes (*FCER1G*, *SYK*, and *PTK2B*) contributing to DPN development using cell and animal models is crucial. Addressing these limitations through large sample‐size omics studies incorporating functional experiments will strengthen the predictive and molecular insights provided by our initial analysis of DPN‐associated molecular patterns.

## 5 CONCLUSION

Immune genes and the associated pathways are crucial in the development of DPN. The activation of monocytes and inactivation of resting NK cells and T follicular helper cells probably aggravate DPN. Three immune genes (*FCER1G*, *SYK*, and *PTK2B*) were identified as biomarkers for DPN diagnosis and progress, serving as potential immunotherapy targets for DPN.

## AUTHOR CONTRIBUTIONS

X.C., Q.L., H.H., and L.Y. conceptualized and designed the study and wrote the manuscript. L.Y. carried out the bioinformatic analyses. X.C., Q.L., N.C., J.M., X.W., and H.Z. contributed through sample collection, experiments, data interpretation, and manuscript preparation.

## FUNDING INFORMATION

This work was supported by a project of Fujian Provincial Health and Health Scientific Research Talent Training (grant number 2019‐ZQN‐66), the Fujian Provincial Natural Science Foundation (grant number 2022J01279), the doctoral research project of The Second Affiliated Hospital of Fujian Medical University (grant number BS202203, 2022BD0704), a project of Fujian Quanzhou Science and Technology Plan (grant number 2020N035s), and the Fujian Provincial Endocrine Clinical Key Specialty Construction Project Funding Project.

## CONFLICT OF INTEREST STATEMENT

None declared.

## Supporting information


**Table S1.** Primmer sequences of genes for RT‐qPCR.
**Table S2.** Summary of biomarkers for diagnosis and prevention of DPN.

## Data Availability

Datasets used in this study were collected from the publicly available GEO database (https://www.ncbi.nlm.nih.gov/geo/).
