# Supplementary material for: Diagnostic biomarker for type 2 diabetic peripheral neuropathy via comprehensive bioinformatics analysis
Source: J Diabetes. 2023 Nov 29;16(3):e13506. doi: 10.1111/1753-0407.13506 (PMC10925884; doi:10.1111/1753-0407.13506)
Supplement: Supplementary file 1 — Table S1. Primmer sequences of genes for RT‐qPCR. Table S2. Summary of biomarkers for diagnosis and prevention of DPN. [file JDB-16-e13506-s001.docx]

# Supplemental tables

**Table S1. Primmer sequences of genes for RT-qPCR.**

| **Gene** | **Forward primer** | **Reverse primer** |
| --- | --- | --- |
| FCER1G | 5'-GCGGGGCTCTCCAGAACATCAT-3' | 5'- AGTCTCGTAAGTCTCCTGGTTCCTG- -3' |
| SYK | 5'-GAAGGCACACCACTACACCATCG-3' | 5'-AGGGCTTCTTGAGGAGGCAGAC-3' |
| PTK2B | 5'-TGGCTGACCTCATAGACGGCTAC-3' | 5'-GCAGGCTGTTCCGCTTCTCAC-3' |
| GAPDH | 5'-GCGGGGCTCTCCAGAACATCAT-3' | 5'-CCAGCCCCAGCGTCAAAGGTG-3' |

Table S2. Summary of biomarkers for diagnosis and prevention of DPN.

| **Biomarker name** | **Type or source** | **AUC** | **References** |
| --- | --- | --- | --- |
| PGP9.5 | Skin punch biopsies | - | Narayanaswamy et al., 2012 |
| TRPV1 | Skin punch biopsies | - |  |
| NSE | Serum | 0.73 | Li et al., 2013 |
| HbA1c | Serum | - | Casadei et al., 2021 |
| Fibrinogen function index k value | Plasma | 0.87 | Zhuang et al., 2022 |
| Fibrinogen function index angle α | Plasma | 0.67 |  |
| TLR4 | Gene | 0.87 | Zhu et al., 2014 |
| TNF-α | Gene | 0.84 |  |
| FCER1G | Gene | 0.84 | This study |
| PTK2B | Gene | 0.73 |  |
| SYK | Gene | 0.81 |  |
